# A community survey on maternal perception about the initiation of dental home for infants in Lagos, Nigeria

**DOI:** 10.11604/pamj.2021.40.78.24441

**Published:** 2021-10-06

**Authors:** Olubukola Olamide Olatosi, Afolabi Oyapero, Gbemisola Ojombo Boyede

**Affiliations:** 1 Department of Child Dental Health, Faculty of Dental Sciences, College of Medicine University of Lagos, Lagos, Nigeria,; 2 Department of Preventive Dentistry, Faculty of Dentistry, Lagos State University College of Medicine, Lagos, Nigeria,; 3 Central North West London National Health Service (NHS), Trust Foundation, London, United Kingdom

**Keywords:** Dental home, first dental visit, maternal perception, practices

## Abstract

**Introduction:**

because efforts directed toward oral health promotion and disease prevention are fundamentally superior to dental rehabilitation after disease development, early preventive dental visits are widely encouraged by dental professional and academic stakeholders. Aim: this study aimed to determine the perceptions and practices of mothers with regards to the establishment of dental home at four local government areas (LGAs) in Lagos, Nigeria.

**Methods:**

was a community-based descriptive household survey conducted amongst mothers in Alimosho, Ikorodu, Surulere and Epe LGAs of Lagos State. Socio-demographic data, information about the importance of primary teeth, knowledge about dental home as well as their child´s age at first dental visit and reasons for attending was obtained with a validated, structured interviewer administered questionnaire. Descriptive statistics, Chi-square and multivariable regression analysis were conducted, and the level of significance was set at P<0.05.

**Results:**

the highest proportion of the mothers were aged between 26-30 years (27.4%; mean age: 34.58±7.8 years) and had a tertiary level of education (n=206, 59.9%); most respondents (n=80, 51.4%) did not know the age a child should be taken to the dental clinic for the first time and had not taken their child for any dental visit (n=229, 65.4%). Out of those who had previously taken their child for dental visits, the greater proportion (n=115, 95.0%) took the child when he/she was older than one year of age. Overall, only 126 (36.0%) respondents had a good perception about oral health and the need for a dental home while 224 (64.0%) respondents had poor knowledge. Logistic regression analysis of predictor variables that showed mothers with a tertiary level of education (OR=0.108; CI=0.0023-0.495) and those with 2-3 children (OR=0.482; CI=0.253-0.920) had significant lower odds of poor perception about the importance of a dental home.

**Conclusion:**

maternal knowledge and practices with regards to dental home were poor and inadequate. It is necessary to create more awareness among parents/caregivers, especially through antenatal and immunization clinics to establish the concept of dental home.

## Introduction

Oral health is multi-faceted and includes the ability to speak, smile, smell, taste, touch, chew, swallow and convey a range of emotions through facial expressions with confidence and without pain, discomfort and disease of the craniofacial complex [1]. Oral disease condition like dental caries, periodontal problems, tooth loss, oro-dental trauma and oro-pharyngeal cancers of the oral tissues and supporting structure of the tooth-related condition are some of the major public health problems worldwide, and poor oral health imposes an enormous impact on general health and quality of life. Oral diseases affect 3.9 billion people globally and have a significant impact on individuals, communities, health systems, economies and society at large. Consequences of oral disease on individuals are both physical and psychosocial. There is a huge burden of untreated oral conditions where the number rose from 2.5 billion in 1990 to 3.5 billion in 2015 which equals to 64% increase of disability adjusted life years lost (DALYs) globally [2]. Yet despite their magnitude, awareness of oral diseases among politicians, health planners and even members of the public health community remains low. This often leads to oral public health interventions to be regarded as a luxury rather than a fundamental human right [3]. Oral diseases disproportionally affect disadvantaged segments of society placing an additional disease burden on these groups. Epidemiological evidence from many diverse countries and different populations has shown that social gradients in oral health outcomes exist [4]. In low- and middle-income countries, dental diseases constitute a neglected epidemic, and rates are increasing [5,6].

Countries differ vastly by economic circumstances, distribution of wealth, availability of technological advances, and even access to basic human needs such as childhood education. Within a population, there are also marked differences in the way individuals live and prosper. All these differences can have an effect on human health, including oral health [7]. Oral disease is the fourth most expensive disease to treat. In high-income countries, the burden of oral disease has been tackled through the establishment of advanced oral-health services which offer primarily treatment to patients. Globally, in many developing countries, access to oral health care services is very limited due to various constraints, and affordability to acquire oral care is very expensive especially in developing countries where dental care insurance is not available [8]. Approaches to improve oral health are relatively ineffective, yet costly [9]. In most low- and middle-income countries, investment in oral health care is low and resources are primarily allocated to emergency oral care and pain relief.

An early-life dental visit can play a fundamental role in maintaining good oral health throughout childhood, as it represents an opportunity to respond to several highly prevalent diseases that impact quality of life, including dental caries, dental trauma, and malocclusion [10,11]. Dental Home is an ongoing relationship between the dentist who is the primary dental care provider and the patient, and it includes comprehensive oral health care, beginning no later than age one. It comprises of all aspects of oral health care delivered in a comprehensive, continuously accessible, coordinated, and family-centered way [12]. Establishing a dental home implies that a child´s oral health care is managed from infancy in with a focus on preventive and early interception of developing dental diseases by a licensed dentist. The range of services provided in the dental home include comprehensive screening for oral diseases, caries-risk assessment, dietary counselling, anticipatory guidance regarding growth and development concerns such as teething and digit sucking habits; as well as oral health education about proper care of the child´s teeth and gingivae. Evidence also indicates that children who receive early preventive dental care are less likely to require subsequent restorative or emergency treatment and have lower dental-related health care costs, particularly among high-risk populations [13]. While the need for dental treatment among Nigerian children is high, there is low and delayed utilization of dental services until oral symptoms such as pain appear and persist [14].

Importantly, early childhood oral health influences and outcomes are considered pivotal in determining oral health trajectories across the life course, and can impact oral health and disease occurrence in adulthood [15]. Little is known about the factors that distinguish families who receive dental care in early childhood from those who do not. However, limited information exists on factors that may modify women´s knowledge and beliefs regarding their infants´ oral health; it is these factors that can influence the development of more cost-effective targeted interventions to improve oral health and timing of children´s entry into the dental care system [16]. This information would assist primary health care providers in targeting preventive care recommendations to families most at risk for not following these recommendations. Given the importance of early preventive dental care, the primary objective of this study was to identify demographic, social, dietary, and biological factors associated with families who do not seek dental care in early childhood in a population.

We hypothesize that maternal knowledge about the age they should take their child for their first dental visit among our study population will not be in line with that recommended by the American Academy of Pediatric Dentistry (AAPD), and that more than half of the population would not have taken their child for the first dental visit by the age of 1 year. Therefore, our study aimed to determine maternal knowledge and practices regarding taking their child for his/her first dental visit before the age of one at four local government areas in Lagos, Nigeria.

## Methods

Before collection of data, ethical approval was obtained for the protocol of this research from the Health Research and Ethics Committee of LUTH at Idi-Araba, Lagos, Nigeria (Protocol Number:ADM/DCST/HREC/APP/2760). Adequate consideration was given to protect the identity of the study participants, and the confidentiality of the information given was guaranteed.

**Study design:** this was a community-based descriptive cross-sectional survey which recruited participants through a household survey.

**Study setting:** Lagos State, located in the South Western of Nigeria and it is the economic hub country. It shares boundaries with Ogun State both in the North and East and on the West by Benin Republic. In the South, it stretches for 180 km along the coast of the Atlantic ocean and occupies an area of 3,577sq km, 22% or 787sq km which consists of lagoons and creeks. It is a commercial nerve center and a cultural melting pot of the diverse population of Nigerians of different cultures and backgrounds, making studies conducted in it more representative than in other cimes in the country. The state is divided into administrative and political geographic units called local government areas (LGAs) and our study was conducted in Alimosho, Ikorodu, Surulere and Epe LGAs of Lagos State.

**Sampling method:** a multi-stage cluster random sampling technique was used for this study. At the first stage, four LGAs were selected out of the 20 LGAs in Lagos State, while four wards were selected in each LGA, using simple random sampling. Stage 2 involved the enumeration of household in the selected wards to create a sampling frame and the selection of eligible households within the enumeration sites. This was done by systematic sampling, with every third household on each street considered eligible for recruitment of a study participant, after a random starting point on the street was determined. At the third stage, one household selected in each compound was randomly recruited and an eligible mother was selected for actual interview after obtaining her informed consent. If there was no eligible mother in the selected household, the next eligible house was selected for recruitment.

**Study participants:** the study population comprised mothers with at least one child, and who had resided in the selected LGAs for at least one year preceding our study, while temporary visitors were excluded. Only mothers who were biological parents or legal guardians and those that were at home at the time of data collection were included in the study.

**Sample size calculation:** the sample size was determined with Leslie Kish´s formula for descriptive surveys. A minimum sample size of 242 participants was calculated, based on the prevalence of poor practices with regards to dental home of 79% from a previous study [14], with a standard normal deviate of 1.96, level of precision set at 5% and a 10% provision made for incomplete responses. We however recruited 350 respondents for the study.

**Data collection tool:** the study was conducted using a previously validated, pretested, structured and interviewer-administered questionnaire. House-to-house survey was conducted from the July to September 2019, by the main researcher and 6 calibrated research assistants who had undergone training for 4 days prior to data collection. The questionnaires were administered on the mother of the child or the primary care-giver. One questionnaire was administered to one household in each compound. Filled questionnaires were crosschecked by the principal researcher for completeness and exactness. The questionnaire was divided into three parts which are section A: this had seven questions on the mothers´ demographic characteristics such as maternal age, religion, ethnic group, educational attainment, work status and number of children in the household and their ages. Section B: obtained information about their perception on the importance of primary teeth, knowledge about dental home and their preference for dental treatment. These included questions on if baby teeth are important, if they think it is necessary to treat baby teeth with decay and the reasons for their responses. It also determined maternal perception on the best treatment for decayed teeth and if untreated tooth decay of deciduous teeth can affect the permanent dentition. It also determined if mothers had received any information on the care of children´s teeth, the source of the information, the age at which a child should be taken to the dental clinic for the first time and if it should be before the first birthday. Section C: comprised of seven questions, which aimed to assess the practices of the mother regarding the child´s first dental visit. It also assessed if their child had been to the dentist before and the reasons for the dental visit. Dental home was recorded as being established if the first dental visit was before the child´s first birthday and if an ongoing relationship between the dentist and the patient/ child pair was established at that visit.

**Data analysis:** the data was entered and analyzed using IBM Statistical Package for the Social Sciences (SPSS) Version 22.0 (IBM Corp, Armonk, NY). Correct answer to each perception question was given a score of one point while a wrong answer was given a score of zero. The overall score for each respondent was computed by adding up all the awarded marks and these were converted to percentages. A knowledge grade was assigned to each respondent based on his total percentage score. In order to dichotomize the variable, the mean of the final scores served as cut-off point, with respondents scoring below the mean categorized as having poor knowledge and all others scoring the mean score and above comprising those with good knowledge. Bivariate and multivariable analyses were carried out. Bivariate analysis was done using Chi-squared test and Fisher's exact test depending on the number of variables in a cell. The mean knowledge score for this sample was 2.24 ± 1.13. For our secondary analysis, we utilized multivariable logistic regression to establish the factors significantly associated with independent predictor variables. Odds ratios (OR) and confidence intervals (CI) were computed for each predictor variable. Level of significance was set at 5% (p≤0.05).

## Results

A total of 395 households were visited, from which 350 respondents consented to participate- a response rate of 88.6%. Table 1 shows that the highest proportion of the mothers were aged between 26-30 years (27.4%) and that their mean age was 34.58 ± 7.8 years. Most mothers were Yoruba (n=199, 56.9%) and had a tertiary level of education (n=206, 59.9%). Majority of them 202 (57.7%) were working full time and had 2-3 children (n=208, 59.4%). Most of them had children below the age of 12 (n=310, 88.6%) with the highest proportion having only one child below 12 years of age (n=116, 37.4%) (Table 1).

**Table 1:** socio-demographic characteristics of respondents

Variable	Frequency (n=350)	Percentage
**Maternal age group (years)**		
≤25	25	7.1
26 - 30	96	27.4
31 - 35	92	26.3
36 - 40	70	20.0
41 - 45	43	12.3
>45	24	6.9
Mean ± SD	34.58 ± 7.8	
**Religion**		
Christianity	239	68.3
Islam	110	31.4
Others	1	0.3
**Ethnic group**		
Yoruba	199	56.9
Igbo	98	28.0
Hausa	29	8.3
Others	24	6.9
**Maternal education**		
None	11	3.1
Primary	19	5.4
Secondary	114	32.6
Tertiary	206	58.9
**Employment status**		
Not working	63	18.0
Part time	70	20.0
Full time	202	57.7
Student	15	4.3
**Number of children**		
1	78	22.3
2 - 3	208	59.4
>3	64	18.3
**Children below 12 years**		
Yes	310	88.6
No	40	11.4
**Number of children below 12 (n=310)**		
1	116	37.4
2	132	42.6
3	47	15.2
4	14	4.5
5	1	0.3

Table 2 shows that 96 percent agreed that baby teeth were important, 179 respondents agreed that untreated baby teeth can affect permanent teeth while 78.9 percent of respondents knew that it was necessary to treat baby teeth with decay. Out of those that felt that it was not necessary to treat baby teeth with decay, the greater proportion (n=60, 81.1%) felt that it was unnecessary to spend time and money on baby teeth. Only 94 respondents (34.1%) stated that the desirable form of treatment for dental complaints was to take the child to the dentist. Table 3 shows that the highest proportion (n=224, 84.0%) of the mother had previously received information about child tooth care and that the antenatal clinic (n=127, 56.7%) was the most common source of information about child oral care. Most respondents (n=180, 51.4%) did not know the age a child should be taken to the dental clinic for the first time and had not taken their child for any dental visit (n=229, 65.4%). Out of those who had previously taken their child for dental visits, the greater proportion (n=115, 95.0%) took the child when he/she was older than one year of age. The most common reason for attendance at the clinic was routine checkup (n=27, 22.3%), followed by dental caries (33.1%, n=383) and dental trauma (n=15, 12.4%).

**Table 2:** maternal perception on the importance of deciduous dentition

Variable	Frequency (n=350)	Percentage
**Baby teeth important**		
Yes	336	96.0
No	14	4.0
**Necessary to treat baby teeth with decay**		
Yes	276	78.9
No	74	21.1
**Reasons for not treating**		
Unnecessary to spend time and money	60	81.1
The children are too small to be treated	14	18.9
**Desirable form of treatment**		
Take to dentist	94	34.1
Hospital	46	16.7
Self-medication and concoction	8	2.9
Brushing teeth with herbs	8	2.9
Filling	47	17.0
Extraction	45	16.3
Don't know	22	8.0
**Untreated baby teeth can affect permanent teeth**		
Yes	179	51.1
No	47	13.4
Don't know	124	35.4

**Table 3:** dental awareness and access to dental care among respondents

Variable	Frequency (n=350)	Percentage
**Previous information about child tooth care**		
Yes	224	84.0
No	126	36.0
*Source of information (n=224)		
Family and relatives	72	32.1
During antenatal clinic	127	56.7
Hospital	88	39.3
Dental clinic	76	33.9
Television	6	2.7
Social media	23	10.3
**Age a child should be taken to the dental clinic for the first time (years)**		
≤1	71	20.3
>1	99	28.3
Don't know	180	51.4
**Has child seen dentist before**		
Yes	121	34.6
No	229	65.4
**Age index child was first taken for dental visit (years) n=121**		
≤1	6	5.0
>1	115	95.0
*Reason for the first visit		
Toothache	11	9.1
Caries	23	19.1
Malocclusion	11	9.1
Trauma	15	12.4
Swelling	11	9.1
Poor oral hygiene	11	9.1
Routine	27	22.3
Good knowledge	126	36.0
Poor Knowledge	224	64.0
Good practices	29	8.3
Poor Practices	321	91.7
*Multiple responses		

Overall, only 126 (36.0%) respondents had a good perception about oral health and the need for a dental home while 224 (64.0%) respondents had poor knowledge. On dichotomizing responses related to practices related to dental care, only 29 (8.3%) respondents had good practices about oral health and dental visits while 321 (91.7%) respondents had poor practices. Table 4 shows the bivariate relationship between socio-demographic characteristics and perception about dental home. There was a statistically significant relationship between maternal level of education (p<0.001) and the number of children in the family (p=0.007) with maternal perception about dental home. Even though the other associations are not significant, younger respondents below 25 years of age (40%), mothers with full time employment (40.6%), and those with children below 12 years of age (36.8%), had a higher proportion of respondents with good perception about dental home.

**Table 4:** bivariate association between socio-demographic characteristics and perception about dental home

	Good (n=126)	Poor (n=224)	X2†	p-value
**Age group (years)**				
≤25	10(40.0)	15(60.0)	4.701	0.621
26 - 30	30(31.2)	66(68.8)		
31 - 35	40(43.5)	52(46.8)		
36 - 40	22(31.4)	48(68.8)		
41 - 45	17(31.4)	25(60.5)		
>45	7(29.2)	17(70.8)		
**Maternal education**				
None	0(0.0)	11(100.0)	22.855	<0.001*
Primary	2(10.5)	17(89.5)		
Secondary	93(45.1)	113(54.8)		
Tertiary	93(45.1)	113(54.9)		
**Employment status**			**0.9136**	**0.288**
Not working	13(20.6)	5(74.9)		
Part time	24(34.3)	46(65.7)		
Full time	82(40.6)	120(59.4)		
Student	7(46.7)	8(53.3)		
**Number of children**				
1	24(30.8)	54(69.20	10.062	0.007*
2 - 3	88(42.9)	120(57.7)		
>3	14(21.9)	50(78.1)		
**Children below 12**				
Yes	114(36.8)	196(63.2)	0.760	0.401
No	12(30.0)	28(70.0)		

* Significant; †Fishers exact when cells were less than 5

Table 5 shows the independent predictors of poor perception about dental home. Logistic regression analysis of predictor variables that showed mothers with a tertiary level of education (OR=0.108; CI=0.0023 - 0.495) and those with 2-3 children (OR=0.482; CI=0.253 - 0.920) had significant lower odds of poor perception about the importance of a dental home. Table 6 shows the bivariate relationship between socio-demographic characteristics of respondents and their dental care practices. There was a statistically significant relationship between maternal level of education (p=0.045), the employment status of the mother (p=0.038) and if they had children in the family below 12 years of age (p=0.043) with maternal practices.

**Table 5:** independent predictors of poor perception

	Odd ratio	95% CI	p-value
**Age group (years)**			
≤25	1		
26 - 30	2.479	0.902 - 6.184	0.079
31 - 35	1.391	0.448 - 3.966	0.537
36 - 40	2.637	0.852 - 8.162	0.093
41 - 45	1.846	0.557 - 6.115	0.316
>45	1.770	0.446 - 7.019	0.417
**Maternal education**			
None/primary	1		
Secondary	0.235	0.051 - 1.094	0.065
Tertiary	0.108	0.023 - 0.495	0.004*
**Employment status**			
Not working	1		
Part time	0.678	0.290 - 1.582	0.368
Full time	0.523	0.277 - 1.223	0.153
Student	0.459	0.128 - 1.649	0.232
**Number of children**			
1	1		
2 - 3	0.482	0.253 - 0.920	0.027*
>3	1.210	0.400 - 2.550	0.984
**Children below 12**			
Yes	1		
No	0.924	0.426 - 2.168	0.924

* Significant; OR: odds ratio; CI: confidence interval

**Table 6:** bivariate association between maternal socio-demographic characteristics and practices

	Good (n=29)	Poor (n=321)	X2†	p-value
**Age group (years)**				
≤25	2(8.0)	23(92.0)	4.840	0.436
26 - 30	11(11.5)	85(88.5)		
31 - 35	10(10.9)	82(89.1)		
36 - 40	3(4.3)	67(95.7)		
41 - 45	2(4.7)	41(95.3)		
>45	1(4.2)	2395.8		
**Maternal education**				
None	0(0.0)	11(10.0)	8.081	0.045
Primary	0(0.0)	19(100.0)		
Secondary	5(4.4)	109(95.6)		
Tertiary	24(11.7)	182(88.3)		
**Employment status**				
Not working	3(4.8)	60(95.2)	8.411	0.038
Part time	4(5.7)	66(94.3)		
Full time	18(8.9)	184(91.1)		
Student	4(26.7)	11(73.3)		
**Number of children**				
1	6(7.7)	72(92.3)	3.176	0.204
2 - 3	21(10.1)	187(89.9)		
>3	2(3.1)	62(96.9)		
**Children below 12**				
Yes	29(9.4)	281(90.6)	4.080	0.043*
No	0(0.00	40(100.0)		

* Significant; †Fishers exact when cells were less than 5

Table 7 shows the independent predictors of poor maternal practices relating to dental care. Logistic regression analysis of predictor variables that showed mothers with a tertiary level of education (OR=0.279; CI=0.095 - 0.818) and those with children below the age of 12 years had significant lower odds of poor practices about dental care.

**Table 7:** independent predictor of poor maternal practices

	Odd ratio	95% CI	p-value
**Age group (years)**			
≤25	1		
26 - 30	1.160	0.199 - 6.774	0.869
31 - 35	1.383	0.215 - 8.887	0.733
36 - 40	3.400	0.392 - 29.513	0.267
41 - 45	3.058	0.294 - 32.904	0.356
>45	1.691	0.109 - 26.234	0.707
**Maternal education**			
None/primary	1		
Secondary	0.453	0.212 - 2.041	0.521
Tertiary	0.279	0.095 - 0.818	0.020*
**Employment status**			
Not working	1		
Part time	1.086	0.217 - 5.433	0.920
Full time	0.773	0.203 - 2.951	0.707
Student	0.285	0.049 - 1.658	0.162
**Number of children**			
1	1		
2 - 3	0.477	0.158 - 1.436	0.188
>3	1.803	0.121 - 5.324	0.820
**Children below 12**			
Yes	1		
No	2.343	1.302 - 4.901	0.023*

* Significant; OR: odds ratio; CI: confidence interval

## Discussion

One of the reasons for unsatisfactory dental health among the youngest population is the delay in the first visit of the child to the dentist. Currently, major professional associations´ (American Academy of Pediatric Dentistry, European Academy of Pediatric Dentistry, American Dental Association, Canadian Dental Association, Australian Dental Association, and American Academy of Pediatrics) recommendations converge to the first dental visit taking place early, at the time of the first tooth eruption (around age 6 months) or by age 1 year [17-21]. Other sources suggest 12-18 months as the optimal time for the first visit [22]. Our results show that 96 percent of mothers agreed that baby teeth were important, 179 respondents agreed that untreated baby teeth can affect permanent teeth while 78.9 percent of respondents knew that it was necessary to treat baby teeth with decay. This was higher than the baseline values obtained in previous Nigerian study [23], where only 54.6% knew that unhealthy deciduous teeth could affect adult dentition, indicating that an increased awareness may have been among mothers have been created by some intervention studies on oral maternal oral health promotion [23,24].

Out of the mothers that felt that it was not necessary to treat baby teeth with decay, the greater proportion (81.1%) felt that it was unnecessary to spend time and money on baby teeth. Only 34% of the respondents stated that the desirable form of treatment for dental complaints was to take the child to the dentist. The general attitude of most parents/caregivers towards the primary dentition is poor, as most parents perceive the primary teeth to be temporary and therefore, do not need treatment unless there is pain [25]. This attitude persists, even though the deciduous dentition is important for mastication, maintaining space for the permanent dentition and guiding them into the occlusion, stimulating normal development of the facial bones and muscles, development of normal speech and contributing to the child´s attractive appearance. Correction of this wrong perceptions should be a continuous target for rectification during oral health promotion session for mothers.

The highest proportion of the mother had previously received information about child tooth care had it at the antenatal clinic (56.7%) primary care providers and pediatricians see children more frequently during early childhood, and have the opportunity to incorporate basic infant oral health care into their practice [26]. A common vision of health with a shared responsibility for children's oral health care among all health care professionals provides the basis for future preventive approaches [26,27]. Some Argentine researchers further argued that the first dental visit of a preventive character should take place about the fourth month of intrauterine life [28]. During this visit the expectant mother receives information about caries, its infectivity, is instructed that the mother is the main source of transmittable streptococcus mutans, and is advised on how to provide oral care to the child and possible preventive procedures. The aim of this visit is to stimulate the interest of the pregnant woman in her own health and that of her unborn child through dental education at the antenatal clinic [28].

Among the main findings of our study was that over 50% of the mothers did not know at what age a child should visit the dentist while more than three-fifth of children had never visited at any time, thus had no opportunity for anticipatory guidance and preventive care. Out of those who had previously taken their child for dental visits, the greater proportion (95.0%) took the child when he/she was older than one year of age. This was only slightly higher than two previous Nigerian studies [14,23] which observed that just 0.8% and 0.5% of their study population took their child for a dental visit before the age of 1 year. In national survey of children's health, a telephone survey conducted on over 100,000 US parents of children from infancy to 17-years-old, found that only 10% of 1-year-old children and 24% of 2-year-olds received a preventive dental visit in the previous year [29]. Various recommendations regarding the timing of children´s first dental visit are available in the public domain [18-21], but despite these, the presence of carious lesions or dental trauma appear to impel most children´s first dental visit [30]. This is further compounding by the inability of caregivers´ to recognize the early signs of dental caries in infants and toddlers [31].

Our study further shows that cumulatively, dental attendance was related to the need for curative rather than preventive care. This further demonstrates that even among children that had a dental visit, receiving preventive and restorative care was surprisingly rare. This shows significant barriers and deficiencies in dental care delivery, indicating that awareness of dental home was not the impetus for the dental visit. Previous studies have similarly shown that the reason for the first dental visit was the need for treatment: as many as 63.12% of all children (one out of three patients) visited the dentist due to pain, cavities or tooth injury. Only 36.8% of patients made their adaptation visit. Very similar findings were presented by Wilk-Sieczak *et al*. [32] who reported that 63% of children made their first dental visit due to the need for treatment (tooth decay, pain). Overall, only 36.0% of our respondents had a good perception about oral health and the need for a dental home while 64.0% respondents had poor knowledge.

Furthermore, we observed striking disparities in perception about dental home and actual dental visitation based on some sociodemographic characteristics of our respondents, as mothers with low educational attainment had lower odds of good perception and practices with regards to dental care. In a previous study, the dental health of five-year-olds had also been highly correlated with maternal levels of education [33]. Mothers, being the primary role model in shaping children´s behavior, have major influences on their children´s oral habits and practices and their educational level is an important socioeconomic indicator that affects the incidence of early childhood caries (ECC) in their children. Hallet and Rourke [34] also showed that the prevalence and severity of ECC is linked to decreasing level of mother´s education. Similarly, mothers with 2-3 children had lower odds of poor maternal perception and practices about dental home while those with more than 3 children had higher odds. Socioeconomic disadvantages has been observed to have a negative impact on dental attendance [35,36]. Researchers observed a strong association between socioeconomic circumstances and caries among young children, with disproportionate rates of caries found among children from deprived backgrounds [37]. Family size may affect health outcomes in individuals and family members as a result of family lifestyle and also because, in larger families, getting material resources needed for the family may disrupt everyday health practices [11]. The establishment of a dental home may thus be especially important for children of caregivers with low health literacy or socioeconomic disadvantage, and those at high risk for dental disease [38].

We aimed to identify the factors that can early influence dental visitation so that we can design cost-effective strategies to enhance early dental access and utilization, since preventive care, early diagnosis, and prompt treatment can address oral diseases less invasively and at a lower cost [39]. The Information presented to parents at the first dental visit will positively shape a positive attitude towards dentistry and motivate them to have more interest in their child´s dental health, and consequently to have regular dental visits to implement preventive procedures and early detection of caries and other dental problems [40].

As confirmed by the findings from our study that showed that most of the mothers that had received information on oral health did so at the antenatal clinic, confirming previous observations that the antenatal clinic is a veritable avenue to promote preventive oral health care and healthy behaviours. Incorporating an oral health component into prenatal services requires coordination between dental providers and other health care providers such as pediatricians, family physicians, midwives, and nurses. Nurses and midwives are, in particular, ideally positioned to promote maternal and child oral health status and to expand their access to preventive dental care especially in deprived populations. Maternity and pediatric nurses can regularly offer expectant mothers with oral health counseling before and after childbirth. They can also play a key role in identifying at-risk mothers or children through oral screening and risk assessment and thus refer them to the dental clinic [41].

A major strength of this study was that it was community-based, rather than being based on a cohort visiting the hospital and participants were selected randomly in a systematic way, thus reducing selection bias. The limitation of the present study includes the descriptive study design, hence it could not establish any temporal relationships nor directly infer a causal relationship. A cross sectional design as utilized in this study could obtain prevalence values based on the descriptive characteristics of the study population but a cohort study design will be required to ascertain the impact of early establishment of dental home on the oral health outcomes of the growing child.

## Conclusion

Maternal knowledge and practices with regards to dental home was poor and inadequate. These disparities were significantly impacted by maternal level of education and family size. It is necessary to create more awareness among parents/caregivers, especially through antenatal and immunization clinics to establish the concept of dental home.

### What is known about this topic

- Oral diseases affect 3.9 billion people globally and have a significant impact on individuals, communities, health systems, economies and society at large;
- Early childhood oral health influences and outcomes are considered pivotal in determining oral health trajectories across the life course, and can impact oral health and disease occurrence in adulthood.

### What this study adds

- Maternal knowledge and practices with regards to dental home were poor and inadequate;
- These disparities were significantly impacted by maternal level of education and family size;
- To improve maternal knowledge and practices, incorporating an oral health component into prenatal services requires coordination between dental providers and other health care providers such as pediatricians, family physicians, midwives and nurses.
